# EGCG, a major green tea catechin suppresses breast tumor angiogenesis and growth via inhibiting the activation of HIF-1α and NFκB, and VEGF expression

**DOI:** 10.1186/2045-824X-5-9

**Published:** 2013-05-02

**Authors:** Jian-Wei Gu, Kristina L Makey, Kevan B Tucker, Edmund Chinchar, Xiaowen Mao, Ivy Pei, Emily Y Thomas, Lucio Miele

**Affiliations:** 1 Cancer Institute, University of Mississippi Medical Center, Jackson, Mississippi 39216, USA

## Abstract

The role of EGCG, a major green tea catechin in breast cancer therapy is poorly understood. The present study tests the hypothesis that EGCG can inhibit the activation of HIF-1α and NFκB, and VEGF expression, thereby suppressing tumor angiogenesis and breast cancer progression. Sixteen eight-wk-old female mice (C57BL/6 J) were inoculated with 10^6 E0771 (mouse breast cancer) cells in the left fourth mammary gland fat pad. Eight mice received EGCG at 50–100 mg/kg/d in drinking water for 4 weeks. 8 control mice received drinking water only. Tumor size was monitored using dial calipers. At the end of the experiment, blood samples, tumors, heart and limb muscles were collected for measuring VEGF expression using ELISA and capillary density (CD) using CD31 immunohistochemistry. EGCG treatment significantly reduced tumor weight over the control (0.37 ± 0.15 vs. 1.16 ± 0.30 g; P < 0.01), tumor CD (109 ± 20 vs. 156 ± 12 capillary #/mm^2; P < 0.01), tumor VEGF expression (45.72 ± 1.4 vs. 59.03 ± 3.8 pg/mg; P < 0.01), respectively. But, it has no effects on the body weight, heart weight, angiogenesis and VEGF expression in the heart and skeletal muscle of mice. EGCG at 50 μg/ml significantly inhibited the activation of HIF-1α and NFκB as well as VEGF expression in cultured E0771 cells, compared to the control, respectively. These findings support the hypothesis that EGCG, a major green tea catechin, directly targets both tumor cells and tumor vasculature, thereby inhibiting tumor growth, proliferation, migration, and angiogenesis of breast cancer, which is mediated by the inhibition of HIF-1α and NFκB activation as well as VEGF expression.

## Introduction

The term ‘green tea’ refers to the product manufactured from fresh tea leaves by steaming or drying at elevated temperatures with the precaution to avoid oxidation of the polyphenolic components known as catechins [1]. The natural product (−)-epigallocatechin-3-gallate (EGCG) accounts for 50-80% of catechins in green tea, representing 200–300 mg in a brewed cup of green tea [2]. Several other catechins such as (−)-epicatechin-3-gallate (ECG), (−)-epigallocatechin (EGC), and (−)-epicatechin (EC) are found in lower abundance in green tea [3]. EGCG is defined as a major green tea catechin that contributes to beneficial therapeutic effects, including anti-oxidant, anti-inflammatory, anti-cancer, and immunomodulatory effects [4-6]. Studies conducted on cell-culture systems and animal models as well as human epidemiological studies show that EGCG in green tea could afford protection against a variety of cancer types [7]. Many studies have shown that EGCG produces anti-cancer effect by modulating the activity of mitogen-activated protein kinases (MAPKs), IGF/IGF-1 receptor, Akt, NFκB and HIF-1α [8-12]. A case–control study including 501 breast cancer cases and 594 controls shows that green tea consumption has a significant trend of decreasing risk in a dose-dependent manner, after adjusting for potential confounding factors [13]. However, the investigations of green tea or EGCG in breast cancer using animal model are very limited, and the role of EGCG in breast cancer therapy is poorly understood.

The growth and expansion of a tumor is mainly dependent on angiogenesis, the formation of new capillaries from pre-existing blood vessels. Avascular tumors are those that do not grow beyond a maximum size of 1 to 2 mm^3^ in the absence of neovascularization, and it may be eliminated by a normal immune system [14]. Angiogenesis requires stimulation of vascular endothelial cells through the release of angiogenic factors. Of these, the vascular endothelial growth factor (VEGF) is the most critical regulator in the development of the vascular system and is commonly overexpressed in a variety of human solid tumors including breast cancer [15]. Cancer cells are under greater hypoxia and oxidative stress than normal cells. Oxygen radicals and hypoxia co-operatively promote tumor angiogenesis [16]. Hypoxia causes the activation of HIF-1, in which it stimulates VEGF expression. HIF-1 levels are also increased by oxygen radicals. In addition, oxygen radicals activate NFκB that also increases VEGF expression. VEGF is a key angiogenic factor that stimulates the growth of tumors including breast cancer, in which VEGF exerts paracrine (especially angiogenesis) and autocrine (proliferation and migration) effects to promote progression of breast cancer [17]. As mentioned above, we believe that EGCG can block highly activated NFκB and HIF-1α pathways in breast tumor. Therefore, we hypothesizes that EGCG directly targets both of tumor cells and tumor vasculature, thereby inhibiting tumor growth, proliferation, migration, and angiogenesis of breast cancer, which is mediated by the inhibition of HIF-1α and NFκB activation as well as VEGF expression. Also, EGCG treatment has no significant effects on the body weight, heart weight, angiogenesis and VEGF expression in normal tissues such as the heart and skeletal muscle.

To test this hypothesis, the present study aimed to determine the following: (a) whether a relative high oral dose of EGCG inhibits tumor growth, tumor angiogenesis, and VEGF expression in an immunocompetent mouse model (C57BL/6) of breast cancer; (b) whether oral EGCG treatment affects angiogenesis and VEGF expression in normal tissues such as the heart and skeletal muscle in the same mice; and (c) whether EGCG inhibits proliferation, migration, VEGF expression, the activation of HIF-1α and NFκB in cultured mouse and human breast cancer cells (E0771, MCF-7 and MDA-MB-231).

## Materials and methods

### Chemicals and cell lines

EGCG was purchased from Sigma Chemical Co. (St. Louis, MO). The mouse breast cancer cells (E0771) which were originally isolated from an immunocompetent C57BL/6 mouse, were provided by Dr. Sirotnak FM at Memorial Sloan Kettering Cancer Center, New York, NY [18]. Human estrogen-receptor positive breast cancer (MCF-7) cells and human triple negative breast cancer (MDA-MB-231) cells were purchased from the American Type Culture Collection (Rockville, MD). All breast cancer cells were maintained as monolayer cultures in RPMI Medium 1640 (GIBCO) supplemented with 10% FBS (HyClone), 100 U/ml penicillin, 100 μg/ml streptomycin, and 0.25 μg/ml amphotericin B, and incubated at 37°C in a humidified 5%CO_2_/air injected atmosphere.

### Animal protocols

The protocols were carried out according to the guidelines for the care and use of laboratory animals implemented by the National Institutes of Health and the Guidelines of the Animal Welfare Act and were approved by the University of Mississippi Medical Center’s Institutional Animal Care and Use Committee. 16 female C57BL/6 mice at 7 weeks of age were purchased from Jackson Laboratory (Bar Harbor, Maine). The mice were allowed to acclimate for 1 week with standard chaw diet (Teklad, Harlan Sprague Dawley; Indianapolis, IN) and tap water before beginning the experiments. The eight week old female mice (n = 16) were inoculated with 10^6 E0771 cells suspended in 100 μl of phosphate-buffered saline into the left fourth mammary gland fat pad. Then, 8 mice received EGCG (25 mg/50 ml) in drinking water for 4 weeks and 8 control mice received drinking water only. Each mouse (20 g) usually drank 2 to 4 ml of water per day. Therefore, EGCG was given around 50 to 100 mg/kg/day to the mice. The body weight of the mice was monitored weekly. Tumor size was monitored every other day in two perpendicular dimensions parallel with the surface of the mice using dial calipers. At the end of the experiment, blood samples, tumors, heart and limb muscles were collected for measuring VEGF expression using ELISA and average microvascular density (AMVD) or capillary density (CD) using CD31 immunohistochemistry.

### Morphometric analysis of angiogenesis in tumor, the heart and limb muscles

The quantification of blood vessels in mouse breast tumor, the heart and limb muscle was determined with the modification of a previously reported method [17,19]. Briefly, the tissues were fixed in 4% neutrally buffered paraformaldehyde. For the heart left ventricular and limb muscle samples, consecutive thin transverse cryosections (5 μm) were cut along the base-apex axis. Consecutive thin cryosections (5 μm) of OCT compound (Sakura Finetek, Torrance, CA) embedded tissue samples were fixed in acetone at 4°C for 10 min. After washing in phosphate-buffered saline (PBS), the sections were treated with 3% H_2_O_2_ for 10 minutes to block endogenous peroxidase activity and were blocked with normal rabbit serum. Then, the sections were washed in PBS and incubated with rat anti-mouse CD31 (PECAM-1) monoclonal antibody (BD Pharmingen, San Diego, CA) at a 1:200 dilution overnight at 4°C. Negative controls were incubated with the rat serum IgG at the same dilution. All sections were washed in PBS containing 0.05% Tween-20, and were then incubated with a 2^nd^ antibody, mouse anti-rat IgG (Vector laboratories, Burlingame, CA) at a 1:200 dilution for 1 hour at room temperature again followed by washing with PBS containing 0.05% Tween-20. The sections were incubated in a 1:400 dilution of Extravadin Peroxidase (Sigma, St. Louis, MO) for 30 min. After washing in PBS containing 0.05% Tween-20, the sections were incubated in peroxidase substrate (Vector laboratories, Burlingame, CA) for 5 min. The sections were washed in PBS containing 0.05% Tween-20 and were counterstained with hematoxylin. A positive reaction was indicated by a brown staining. The microvascular vessels were quantified by manual counting under light microscopy. A microscopic field (0.7884 mm^2^) was defined by a grid laced in the eye-piece. At least 20 microscopic fields were randomly acquired from each tumor for analysis. Any endothelial cell or cell cluster showing antibody staining and clearly separated from an adjacent cluster was considered to be a single, countable microvessel. The value of average microvascular density (AMVD) or capillary density (CD) was determined by calculating the mean of the vascular counts per mm^2^ obtained in the microscopic fields for each tissue sample.

### Measurements of protein levels of VEGF by ELISA

Protein levels of VEGF in plasma, breast tumor, the heart, the limb muscle, and the medium cultured with E0771 cells were determined using mouse VEGF ELISA kits (R&D Systems, Minneapolis, MN), according to the manufacturer’s instructions. The total proteins of breast tumor, the heart, the limb muscle, and cultured E0771 cells were extracted using NE-PER Cytoplasmic Extraction Reagents (Pierce, Rockford, IL), according to the manufacturer’s protocol. The total protein concentration of these tissue extractions was determined using a Bio-Rad Protein Assay (Bio-Rad Laboratories, Hercules, CA). The protein concentrations of VEGF were normalized and expressed as pictograms per milligram of total tissue or cell extraction protein.

### Proliferation assay of cultured breast cancer cells

The E0771, MCF-7, and MDA-MB-231cells were seeded into 6-well tissue culture plates using RPMI Medium 1640 (GIBCO) supplemented with 10% FBS (HyClone), 100 U/ml penicillin, 100 μg/ml streptomycin, and 0.25 μg/ml amphotericin B, and incubated at 37°C in a humidified 5%CO_2_/air injected atmosphere. When the monolayer reached about 80% confluence, the cells were washed with PBS and incubated with fresh RPMI Medium 1640 with 10% FBS in the absence and presence of EGCG (0, 10, 50 μg/ml) for 18 hours. 3H-thymidine incorporation assay was used to determine the cell proliferation during the last 6 hours of incubation as previously described [20].

### Migration assay

Migration was determined using BD BioCoat Matrigel Invasion Chamber (BD Bioscience Discovery Labware, Sedford, MA) according to a previous study, in which only invasive cells digested the matrix and moved through the insert membrane [21]. 1 × 10^5^ E0771 cells per well in 0.5 ml medium (RPMI Medium 1640) were seeded in the matrigel-coated upper compartment (insert) of a Transwell (24-well format, 8-μm pore) in the absence of and presence of EGCG (0, 10, 20, 50 μg/ml) and the medium with 10% FBS was added to the lower part of the well. After overnight incubation at 37°C and 5% CO_2_, cells on the upper surface of the insert were removed using a cotton wool swab. Migrated cells on the lower surface of the insert were stained using DiffQuit (Dada Behring, Düdinen, Switzerland). The images of migrated cells were taken and the number of migrated cells was counted using a microscope (Leica, Germany) in a 20× objective.

### HIF-1α and NFκB activation (motif binding) assays

We determined HIF-1α and NFκB activation in cultured E0771 cells in the absence and presence of EGCG (0 and 50 mg/ml) to investigate whether the down-regulation of VEGF by EGCG is associated with the inhibition of HIF-1α and NFκB activation (n = 6). The nuclear proteins were extracted by using Active Motif (Carlsbad, CA) nuclear extract kit. 20 μg nuclear proteins from each sample was used in the TransAM HIF-1α or NFκB p65 kit (Active Motif), which can measure the binding of activated HIF-1α or NFκB to its consensus sequence attached to a microwell plate, according the manufacturer’s instructions.

### Statistical analysis

All determinations were performed in duplicated sets. Where indicated, data is presented as mean ± SE. Statistically significant differences in mean values between the two groups were tested by an unpaired Student’s t-test. Linear regression was performed by the correlation analysis between two continuous variables. A value of P < 0.05 was considered statistically significant. All statistical calculations were performed using SPSS software (SPSS Inc., Chicago, IL).

## Results

### A relative high oral dose of EGCG significantly inhibits the progression of breast cancer growth

**Figure 1:**
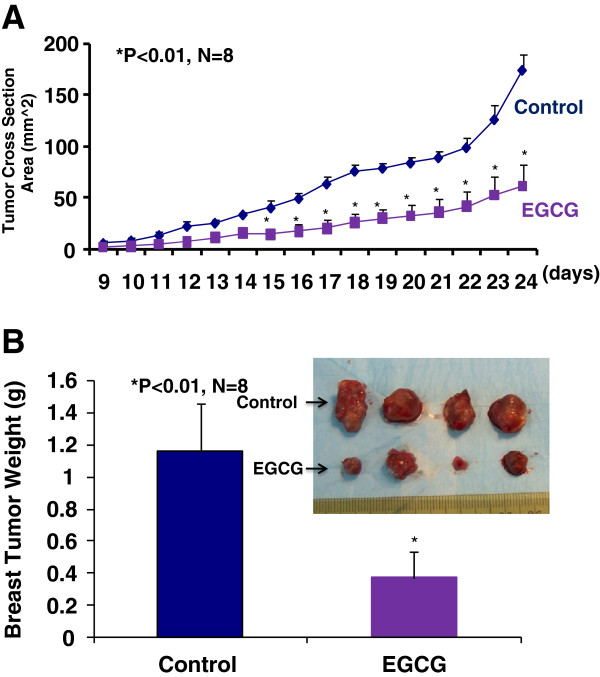
**The inhibition of the progression of breast cancer growth by oral EGCG in the immunocompetant female mice (C57BL/6) allografted with mouse breast cancer (E0771) cells.** EGCG at 50 to 100 mg/kg/day in drinking water for four weeks significantly reduced a growth curve of breast cancer monitored by the tumor cross section area by 65% (Figure 1A, P < 0.01; n = 8) and tumor weight (Figure 1B, 0.37 ± 0.15 vs. 1.16 ± 0.30 g; P < 0.01; n = 8), compared to the control group.

We used a mouse breast cancer model that mimics the human disease, in which the mouse breast adenocinoma (E0771) cells were injected into the pad of the fourth mammary gland of female immunocompetent mice (C57BL/6). Immediately after the inoculation of E0771 cells, the eight week old female mice (n = 8) were given EGCG at 50 to 100 mg/kg/day in drinking water for four weeks and the control group (n = 8) was given regular drinking water only. Tumor size was then monitored every other day in two perpendicular dimensions parallel with the surface of the mice using dial calipers. As indicated in Figure 1A, the tumor cross section area was significantly reduced in the EGCG-treated group compared to the control group two weeks after the breast cancer inoculation. At the end of experiment, the tumor cross section area was reduced by 65% (P < 0.01) in EGCG-treated group compared to the control group (Figure 1A), which was consistent with the reduction in tumor weight (Figure 1B) in EGCG-treated group compared to the control group (0.37 ± 0.15 vs. 1.16 ± 0.30 g; P <0.01). Clearly, EGCG treatment at 50 to 100 mg/kg/d in drinking water significantly inhibited the progression of breast cancer growth in the female mice by decreasing the tumor size and reducing the growth curve of breast cancer. However, there was no significant difference in the body weight, heart weight, kidney weight, or urinary protein between the EGCG-treated mice and the control mice.

### EGCG suppresses breast tumor angiogenesis and VEGF expression in mice

**Figure 2:**
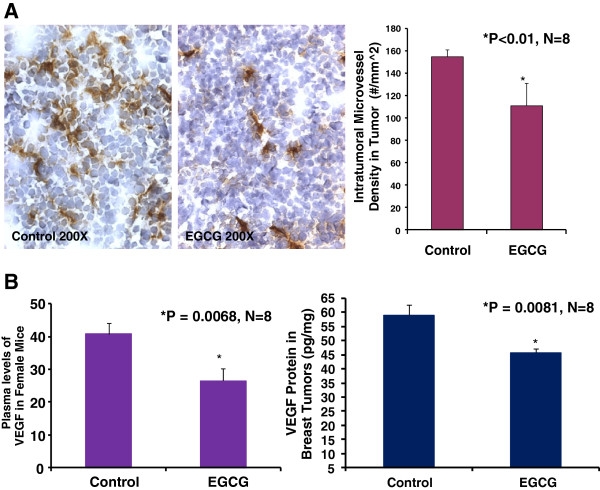
**Oral EGCG at 50–100 mg/kg/d in drinking water significantly reduced intratumoral microvessel density (Panel A: 109 ± 20 vs. 156 ± 12 microvessel #/mm^2; P < 0.01), plasma VEGF levels (Panel B; 26.48 ± 3.76 vs. 40.79 ± 3.5 pg/ml; P < 0.01), and tumor VEGF expression (Panel B; 45.72 ± 1.4 vs. 59.03 ± 3.8 pg/mg; P < 0.01) over the control, respectively in mice (n = 8).** The digital images show CD31 immunohistochemistry staining in OCT-embedded cryosections of mouse breast cancer tumors obtained from a control (Figure 2A) or EGCG-treated (Figure 2A) mouse.

Growth and expansion of tumor mass are strictly dependent on angiogenesis because neovascularization permits rapid tumor growth by providing an exchange of nutrients, oxygen, and paracrine stimuli to the tumor [22]. Therefore, in this study, we used a morphometric analysis of immunohistochemical staining for CD31 to determine the effect of EGCG on breast tumor angiogenesis in mice. Representative images of CD31 staining of the breast cancer tumors showed that the EGCG-treated tumor had lesser microvessels than the control tumor (Figure 2A). Morphometric analysis (Figure 2A) indicated that PDTC treatment caused a significant decrease in average microvessel density (AMVD, the number of microvessels per mm2 area) of breast tumors compared to the control breast tumors (109 ± 20 vs. 156 ± 12 microvessels number per mm^2; n = 8; P < 0.01). These results also suggest that a pronounced decrease in tumor angiogenesis is associated with a decrease in tumor size of breast cancer tumor in the female mice treated with EGCG compared to those in the control mice. Figure 2B also demonstrated that EGCG treatment reduced plasma VEGF levels over the control mice (26.48 ± 3.76 vs. 40.79 ± 3.5 pg/ml; n = 8; P < 0.01) and tumor VEGF expression over the control mice (45.72 ± 1.4 vs. 59.03 ± 3.8 pg/mg; n = 8; P < 0.01). These findings suggest that the inhibition of tumor angiogenesis in mice by EGCG is due to the down-regulation of VEGF because VEGF is a key angiogenic factor.

### EGCG directly inhibits proliferation and migration of breast cancer cells

**Figure 3:**
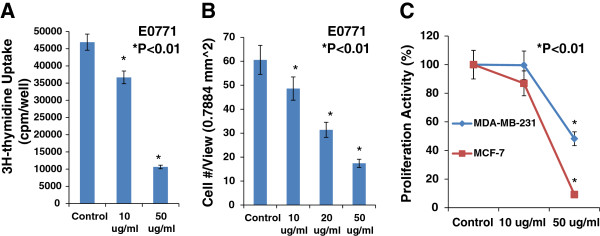
**EGCG caused a dose-related inhibition in 3H-thymidine incorporation, decreasing by 22% at10 μg/ml and by 77% at 50 μg/ml (Panel A, n = 6, P < 0.01), and in migration (Panel B, n = 6, P < 0.01) in cultured E0771 cells, compared to the control group.** In Panel **C**, EGCG at 50 μg/ml significantly inhibited the proliferation in cultured MCF-7 and MDA-MB-231 cells, compared to the control group (n = 6; P < 0.01), respectively.

We used a 3H-thymidine incorporation assay to determine the effects of EGCG on the proliferation of cultured mouse breast cancer cells (E0771), human estrogen receptor positive breast cancer cells (MCF-7), and triple negative breast cancer cells (MDA-MB-231). Figure 3A showed that E0771 cells treated with EGCG caused a dose-related decrease in 3H-thymidine incorporation, decreasing by 22% at 10 μg/ml and by 77% at 50 μg/ml, compared to the control group (n = 6; P < 0.01). We examined the inhibitory effect of EGCG on E0771 cell migration using BD BioCoat Matrigel Invasion Chamber. Figure 3B demonstrates that EGCG at 10, 20, and 50 μg/ml caused a dose-dependent reduction of migrated breast cancer (E0771) cells, decreasing by 25%, 48%, and 71%, respectively, compared to the control group (n = 6; P < 0.01). In the another experiment, as shown in Figure 3C, we demonstrated that EGCG at 50 μg/ml significantly inhibited the proliferation of human estrogen receptor positive breast cancer cells (MCF-7) and triple negative breast cancer cells (MDA-MB-231) by 91% and 52%, respectively, compared to the control group (n = 6; P < 0.01), but not at 10 μg/ml. These *in vitro* findings illustrate that EGCG can directly target breast cancer cells by inhibiting the proliferation and migration.

### The down-regulation of VEGF expression by EGCG is associated with the inhibition of HIF-1α and NFκB activation

**Figure 4:**
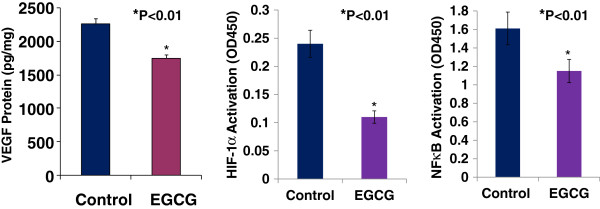
EGCG at 50 μg/ml significantly inhibited VEGF expression (Panel A, 1752 ± 49 vs. 2254 ± 91 pg/mg, n = 6, P < 0.01), the activation of HIF-1α (Panel B, 0.11 ± 0.02 vs. 0.24 ± 0.02, n = 6, P < 0.01) and NFκB (Panel C, 1.15 ± 0.21 vs. 1.61 ± 0.32; n = 6, P < 0.01) in cultured E0771 cells, compared to the control, respectively.

HIF-1 and NFκB pathways are highly activated in breast tumor, in which they can co-operatively promote tumor angiogenesis by increasing VEGF expression [16]. We used VEGF ELISA kit and HIF-1α and NFκB activation (Motif Binding) assays to determine whether EGCG could suppress HIF-1α and NFκB activation and VEGF expression in cultured mouse breast cancer (E0771) cells. Figure 4A showed that EGCG at 50 μg/ml significantly inhibited VEGF expression (1752 ± 49 vs. 2254 ± 91 pg/mg; n = 6; P < 0.01) in cultured E0771 cells, compared to the control. In the same experiment, EGCG at 50 μg/ml also significantly suppressed the activation of HIF-1α (0.11 ± 0.02 vs. 0.24 ± 0.02; P < 0.01; Figure 4B) and NFκB (1.15 ± 0.21 vs. 1.61 ± 0.32; n = 6; P < 0.01; Figure 4C), compared to the control, respectively. These results suggest that the inhibition of HIF-1α and NFκB activation contributes to the down-regulation of VEGF expression.

### Oral EGCG treatment has no effects on angiogenesis and VEGF expression in normal tissues such as the heart and skeletal muscle in mice

**Figure 5:**
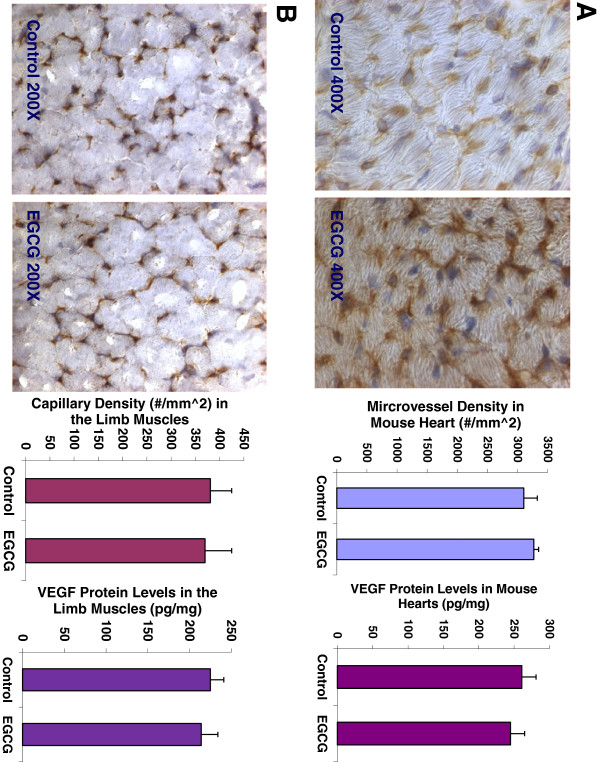
**EGCG treatment did not affect the capillary density (3270 ± 162 vs. 3103 ± 226 #/mm^2; n = 8; P = 0.5215), and VEGF expression (261 ± 22 vs. 245 ± 19 pg/mg; n = 8; P = 0.4517) in the mouse heart, compared to the control group (Panel A), respectively.** There was no significant difference in the capillary density (370 ± 55 vs. 381 ± 44 #/mm^2; n = 8; P = 0.5401), and VEGF expression (225 ± 16 vs. 214 ± 20 pg/mg; n = 8; P = 0.7825) in the limb skeletal muscles between the EGCG-treated mice and the control mice (Panel **B**), respectively. The digital images show CD31 immunohistochemistry staining in OCT-embedded cryosections of the heart (Panel **A**) and the limb muscle (Panel **B**) of control mouse and EGCG-treated mouse, respectively.

The data showed that there was no significant difference in the body weight (22.38.25 ± 0.51 vs. 22.94 ± 0.57; n = 8; P = 0.9437), heart weight (84.7 ± 11.2 vs. 85.1 vs. 10.6 mg; n = 8; P = 0.3546), or kidney weight (237.5 ± 9.2 vs. 240.1 ± 8.9 mg; n = 8; P = 0.3735) between the EGCG-treated mice and the control mice. Figure 5A showed that EGCG treatment did not affect the capillary density (number of capillary/mm^2 area) (3270 ± 162 vs. 3103 ± 226 #/mm^2; n = 8; P = 0.5215) analyzed by CD31 immunochemistry and morphometric analysis, and VEGF expression (261 ± 22 vs. 245 ± 19 pg/mg; n = 8; P = 0.4517) determined by ELISA in the mouse heart, compared to the control group, respectively. Figure 5B showed that there was no significant difference in the capillary density (370 ± 55 vs. 381 ± 44 #/mm^2; n = 8; P = 0.5401), and VEGF expression (225 ± 16 vs. 214 ± 20 pg/mg; n = 8; P = 0.7825) in the limb skeletal muscles between the EGCG-treated mice and the control mice, respectively. These findings illustrate that EGCG does not significantly affect angiogenesis and VEGF expression in the normal tissues such as the heart and skeletal muscles.

## Discussion

The major new findings from this study include: 1) a relative high oral dose of EGCG significantly inhibits the progression of mouse breast cancer growth in female immunocompetent mice; 2) EGCG significantly suppresses breast tumor angiogenesis and VEGF expression in these mice; 3) EGCG treatment does not significantly affect angiogenesis and VEGF expression in the normal tissues such as the heart and skeletal muscles in the same experiment; 4) EGCG directly inhibits proliferation and migration of cultured mouse and human breast cancer cells; and 5) the down-regulation of VEGF expression by EGCG is associated with the inhibition of HIF-1α and NFκB activation. These findings support the hypothesis that EGCG, a major green tea catechin directly targets both of tumor cells and tumor vasculature, thereby inhibiting tumor growth, proliferation, migration, and angiogenesis of breast cancer, which is mediated by the inhibition of HIF-1α and NFκB activation as well as VEGF expression. Also, EGCG treatment has no significant effects on angiogenesis and VEGF expression in normal tissues such as the heart and skeletal muscle.

An important finding of this study is that a relative high oral dose of EGCG treatment at 50 to 100 mg/kg/day in drinking water significantly slows a growth curve of breast cancer in C57BL/6 female mice compared to the control group, which is characterized by 65% and 68% reduction in the tumor cross section area and tumor weight, respectively. Clearly, oral EGCG treatment is very effective in suppressing progression of breast cancer in a wild type immunocompetent mouse model. Ullmann et al. reported that peak plasma concentrations were greater than 3 μg/ml after oral dose of 1600 mg in healthy human subjects [23]. We believe that oral dose of 50 to 100 mg/kg/day in human can reach the effective plasma concentrations of EGCG against breast cancer. Recent methods developed for the stereoselective total synthesis of EGCG, and structurally related catechins, could provide new sources of these compounds for biomedical use [24]. Our next step is clinical trial for EGCG in breast cancer therapy.

Cancer cells are under greater hypoxia and oxidative stress than normal cells. 8-hydroxy-2’-deoxyguanosine, a major marker of constitutive oxidative stress is almost 10 times more prevalent in invasive ductal breast carcinoma cells than in normal control samples from the same patient [25]. Tumor cells overproduce reactive oxygen species (ROS) by alterations to metabolic pathways in tumor cells [26], an inadequate tumor vascular network [16], and macrophage infiltration of the tumor [27]. Breast carcinomas support their growth by stimulating angiogenesis. Blood flow within these new vessels is often chaotic, causing periods of hypoxia followed by reperfusion. The generation of ROS by reperfusion further causes oxidative stress within breast carcinomas. Also, a breast carcinoma rapidly outgrows its blood supply, leading to glucose deprivation and hypoxia. Glucose deprivation rapidly induces oxidative stress within breast carcinoma cells [28]. Clearly, hypoxia and oxidative stress are found together within the breast carcinoma, in which VEGF production can be augmented by synergy between oxygen radicals and tumor hypoxia. Oxygen radicals and hypoxia co-operatively promote tumor angiogenesis [16]. Hypoxia causes the activation of HIF-1, in which it stimulates VEGF expression. HIF-1 levels are also increased by oxygen radicals. In addition, oxygen radicals activate NFκB that also increases VEGF expression. Thus, the compound blocking HIF-1and NFκB pathways can significantly inhibit VEGF expression and angiogenesis in carcinomas including breast carcinomas.

In this study, we found that the significant inhibitions of tumor growth and tumor angiogenesis of breast cancer in female mice by EGCG were associated with suppressing the activation of HIF-1α and NFκB, and decreasing VEGF expression in breast carcinoma cells. VEGF is a key angiogenic factor that stimulates the growth of tumors including breast cancer, in which VEGF exerts paracrine (especially angiogenesis) and autocrine (proliferation and migration) effects to promote progression of breast cancer [17]. VEGF overexpression and the activation of HIF-1α and NFκB pathways in breast cancer are strongly linked to rapid growth of tumors and worse prognosis [16,29,30]. Oxygen radicals and hypoxia co-operatively promote tumor angiogenesis, in which VEGF overexpression is stimulated by the activation of HIF-1α and NFκB pathways in breast cancer [16]. The present findings indicate that EGCG significantly inhibits VEGF expression by suppressing the activation of HIF-1α and NFκB pathways, thereby inhibiting tumor growth, proliferation, migration, and angiogenesis of breast cancer. Our results are supported by the previous findings as follows: 1) EGCG suppressed tumor growth by blocking the induction of VEGF in human colon carcinoma cells [31]; 2) EGCG inhibited VEGF/VEGFR axis by suppressing the expression of HIF-1α in human colorectal cancer cells [32]; and 3) EGCG inhibited cancer progression by decreasing NFκB activation [33]. Progression stage is the final phase of cancer development, an uncontrolled growth of cancer cells occurs. In this stage cancer cells are under greater hypoxia and oxidative stress, in which many transcription factors, such as HIF-1α and NFκB, are activated leading to transmit aberrant signals resulting in abnormal functions such as tumor angiogenesis, cancer invasiveness and metastasis. Present findings illustrate that EGCG can inhibit multiple key cellular signals resulting in inhibiting tumor angiogenesis and breast cancer progression. Also, accumulating evidence shows that EGCG can target all stages of cancer development by blocking multiple cellular proteins involved in diverse cellular signal transduction pathways: proliferation, differentiation, apoptosis, angiogenesis or metastasis [34]. In future study, we will investigate the therapeutic potentials of EGCG combined with VEGF receptor inhibitor, Notch inhibitor, HIF-1 inhibitor, or NFκB blocker in breast cancer therapy.

In present study, we demonstrated that EGCG treatment reduced plasma VEGF levels by 35% over the control mice, which was associated with more than 65% reduction of tumor weight in EGCG treated breast cancer mice, compared to untreated breast cancer mice. These findings are consistent with breast cancer patients that EGCG treatment reduced serum levels of VEGF [35]. A study on 200 women showed that serum VEGF levels were significantly higher in breast cancer patients compared to control [36]. Systemic VEGF levels were reduced significantly in the breast cancer patients following tumor excision [36]. We believe that oral EGCG treatment could reduce the tumor-related blood VEGF levels.

Interestingly, the present study shows that EGCG treatment does not significantly affect angiogenesis and VEGF expression in the normal tissues such as the heart and skeletal muscles in the same experiment. The present study first time shows that oral EGCG treatment significantly inhibits angiogenesis, VEGF expression, and growth in breast tumor, but no such effects on the normal tissues such as the heart and limb muscles in the same mice. The different effects of EGCG in tumor and normal tissues can be explained by that cancer cells are under greater hypoxia and oxidative stress than normal cells. VEGF expression and angiogenesis are very stable in normal matured tissues in which they are regulated by metabolic balance within the tissue. However, angiogenesis is stimulated by significantly increased VEGF levels, activated HIF-1α and NFκB pathways in cancer. We also found that there was no significant difference in the body weight, heart weight, or kidney weight between EGCG-treated mice and the control mice. This is an exciting possibility, because EGCG is a drug of low toxicity.

Antiangiogenic therapy is an attractive approach for cancer treatment including breast cancer, in which these agents include monoclonal antibodies (mAbs) and the tyrosine kinase inhibitors (TKIs) of VEGF pathway. Implicated in many physiological processes, VEGF pathway inhibition can lead to on-target side effects, such as hypertension, proteinuria, thromboembolic events, or congestive heart failure [37-39]. The incidence of hypertension rate was up to 35% with bevacizumab, a monoclonal antibody against VEGF-A [40,41]. Ultimately, considering the modest clinical benefit on the one hand, and the increase in toxicity on the other, the US Food and Drug Administration withdraw its approval of the breast cancer treatment for bevacizumab [42]. As mentioned above, EGCG is a drug of low toxicity, and significantly inhibits angiogenesis in breast tumor (under greater oxidative stress), but not in the normal tissues (no oxidative stress) such as the heart and limb muscles in the same mice. Thus, EGCG may overcome the existing barriers - the mAbs and TKIs of VEGF pathway-induced on-target side effects. However, the further studies are needed.

In conclusion, our results indicate that oral administration of EGCG, a major green tea catechin, significantly inhibits tumor growth and tumor angiogenesis of breast cancer, but no effect on angiogenesis in the heart and limb muscles in an immunocompetent mouse model using mouse breast cancer (E0771) cells. EGCG directly suppresses the proliferation or migration of cultured mouse breast cancer cells as well as the proliferation of human breast cancer cells (MCF-7 and MDA-MB-231). These anticancer effects of EGCG seem to be mediated by blocking multiple intracellular signaling cascades such as HIF-1α and NFκB pathways. The mechanistic advance of EGCG on inhibiting tumor angiogenesis is very unique, in which EGCG does not target angiogenesis in normal tissue. Accumulating evidence indicates that EGCG displays a vast array of cellular effects involved in all stages of cancer development. The multiple targets on cancer and less side effects of EGCG will lead a successful targeted therapy for cancers including breast cancer. The potential therapeutic targets of EGCG in cancer therapy are needed to be further explored. Our next step is clinical trial for EGCG in breast cancer therapy. The combination of EGCG with other targeted compounds such as VEGF receptor inhibitor, Notch inhibitor or HIF-1 inhibitor could lead to a very effective specific targeted breast cancer therapy.

## Competing interests

The authors declare that they have no competing interests.

## Authors’ contributions

JG prepared the manuscript and all figures. KM did the animal studies. KT measured capillary density using CD31 immunohistochemistry. EC performed ELISA and proliferation assays. XM prepared discussion for the manuscript. IP worked on migration assay. ET did HIF-1α and NFκB activation assays. LM edited the final manuscript. All authors read and approved the final manuscript.
